# Factors associated with dog ownership and contact with dogs in a UK community

**DOI:** 10.1186/1746-6148-3-5

**Published:** 2007-04-03

**Authors:** Carri Westgarth, Gina L Pinchbeck, John WS Bradshaw, Susan Dawson, Rosalind M Gaskell, Robert M Christley

**Affiliations:** 1 Department of Veterinary Clinical Science, University of Liverpool, Leahurst, Neston, Cheshire, CH64 7TE, UK; 2 Veterinary Pathology, University of Liverpool, Leahurst, Neston, Cheshire, CH64 7TE, UK; 3 Anthrozoology Institute, Department of Clinical Veterinary Science, University of Bristol, Langford House, Langford, North Somerset, BS40 5DU, UK

## Abstract

**Background:**

Dogs are popular pets in many countries. Identifying differences between those who own dogs or have contact with dogs, and those who do not, is useful to those interested in the human-animal bond, human health and for provision of veterinary services. This census-based, epidemiological study aimed to investigate factors associated with dog ownership and contact with dogs, in a semi-rural community of 1278 households in Cheshire, UK.

**Results:**

Twenty-four percent of households were identified as dog-owning and 52% owned a pet of some type. Multivariable logistic regression suggested that households were more likely to own a dog if they had more occupants (five or more); if they had an adult female household member; or if they owned a horse. The age structure of the households was also associated with dog ownership, with households containing older children (between six and 19 years of age) and young adults (between 20 and 29 years of age), more likely to own dogs. We also found that dog owning households were more likely to be multi-dog households than single-dog if they also owned a cat or a bird, or if the household contained a person of 20–29 years old. Dog owners reported increased contact with dogs, other than their own, compared to those that did not own dogs and this contact appeared to be mainly through walking.

**Conclusion:**

Some household types are more likely to own a dog than others. This study supports the suggestion that dogs are more common in families who have older children (6–19 years), as has been generally observed in other countries. Dog owners are also more likely to have contact with dogs other than their own, compared with those not owning a dog.

## Background

Humans and dogs have lived in close proximity for thousands of years. The effect of pet ownership on human health has been studied on a number of occasions but has been somewhat inconclusive due to the difficulties in studying such a complex relationship and assigning direction of causation [1]. Pets provide companionship and also probably confer physiological health and psychological benefits [1-5]. For example, pet owners have fewer doctors visits [6] and longer survival following heart attack [7], compared to non-pet owners. Katcher and Friedmann [4] suggested seven common functions of pet ownership: companionship; something to care for; something to touch and fondle; something to keep one busy; a focus of attention; exercise and safety. Pets have also been indicated to have important roles in enhancing child development [4,8,9], the wellbeing of older people [4,9,10] and may also be used in a therapeutic setting [8,10,11]. However, it has become increasingly apparent that pets are an important source of zoonotic infections. Approximately 30 to 40 organisms that cause zoonotic infections are known in companion animals, including dogs [12]. Some groups in human society are at greater risk of zoonotic infection due to their immune system or behaviour, for example young children, the elderly, pregnant women and the immunocompromised [12,13].

Virtually everyone in the community is in contact with either companion animals or their products, including excreta [14]. Dogs and cats are the most popular pets in the UK, although dog ownership has declined slightly over recent years [15]. In 2004 there were approximately 5.2 million dog owning households in the UK (21% of households), owning 6.8 million dogs [15]. The demographics of pet ownership are of health, psychological and social science interest, applicable to the research area of the nature of the pet-human bond and can also be used to inform provision of veterinary services.

Previous studies in the USA and Australia have suggested that pets are more common in families who have children [16-26]. In contrast, a study of dog ownership in Germany found that the majority of the dog owners did not live with children younger than 18 years of age [27]. This project aimed to investigate factors associated with dog ownership in a semi-rural community using a doorstep interview questionnaire. The previous studies mentioned used telephone interview or mail questionnaire methods to sample a small proportion of a large study population. In contrast, this study attempted to doorstep interview all households in a defined geographic area to produce a detailed census of a single community. Whereas some other studies have combined dogs and cats as 'pets' for analysis, this study focused specifically on dogs; reasons for dog versus cat ownership are likely to differ as they have different ownership requirements.

## Results

A total of 1142 households (89% of all council registered households) in the study area were contacted within five visits to the property. A further 136 households (11%) could not be contacted during five attempts, although 2% of the total properties were suspected to be unoccupied. Over half (53%) of the households were interviewed during the first round of visits. Of those households contacted and asked to participate, 1051 (92%) were fully interviewed, 24 (2%) part-interviewed (answered some but not all questions) and 67 (6%) were not willing to participate in the study. This gave an overall usable response rate of 84%.

Of the households contacted, 24% (266) were identified as dog owning (DO); only four of these DO households were not willing to be interviewed. Two hundred and one (77%) DO households owned one dog, 53 (20%) two dogs and eight (3%) three dogs (mean 1.3 dogs). Just over half (52%) of interviewed households owned a pet of some type. A variety of other pets were identified, cats (22% of households) being the most popular after dogs.

The most common reason given for not owning a dog by dog-free households (DF) was due to 'working or being out all day' (26% households) followed by 'not enough time for a dog' (15%). 'Do not like dogs' was reported less commonly (10%). Sixty-two percent of interviewees who did not own a dog had owned one in the past (including as a child). In such cases, the last dog had been owned a median of 10 years previously (interquartile range 5–24 years) with a maximum of 80 years previously. Households owning a dog reported 'companionship' (68%) and 'always had a dog' (42%) as their most common reasons for owning a dog.

**Figure 1:**
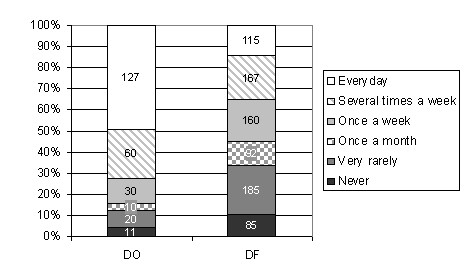
**Reported contact with dogs (other than own dog) for Dog-owning (DO) and Dog-free (DF) households**. The numbers indicate the number of respondents in each category. DO households were significantly more likely to have more frequent contact with dogs, compared to DF households (P < 0.001).

When asked how often they came into physical contact with dogs (other than their own) DO reported increased contact compared to DF (P < 0.001): 'Everyday' (DO vs. DF; 49% vs. 14%) was clearly the most common answer from dog owners, whereas 'several times a week' (23% vs. 21%), 'once a week' (12% vs. 20%), or 'very rarely' (8% vs. 23%) were more common responses for those not owning a dog themselves (Figure 1). Interviewees were asked to suggest circumstances in which they come into contact with dogs other than their own; the most common answers were 'friends' (32%), 'walking' (31%) and 'family' (29%). DO respondents reported increased contact whilst walking (OR = 7.4, 95%CI 5.4–10.0) compared to DF respondents. Other effects of dog ownership included decreased contact with dogs through neighbours and increased contact through employment (OR = 0.6, 95%CI 0.4–1.00 and OR = 1.8, 95%CI 1.1–2.9 respectively).

### Univariable analysis

Univariable analysis of DO versus DF (Table 1) identified presence of birds, fish and horse as significantly positively associated with DO status (P < 0.05). There was no evidence of an association between cat ownership and owning a dog.

There was no evidence for a significant effect of either house type (flat, terrace, semi-detached and detached) or street type (cul-de-sac, through road) on household ownership of dogs (P = 0.6 and P = 0.9 respectively) in this area. There was an insufficient number of households with no garden or only a yard to compare with those with a garden for analysis of this factor. There was no evidence for a significant difference between DO and DF with respect to the amount that the garden was used for recreational purposes (such as eating, gardening, children playing) (P = 0.98). The most common response was 'often' (74%), possibly due to the study being conducted during summer months.

Two person households were most common in this population (37%). DO households were associated with a greater numbers of persons living in them (Table 1). The median number of persons per household was two for DF and three for DO, both with interquartile ranges 2–4. Mixed adult gender households were more likely to own a dog than single gender households. However this variable was associated with the number of people in the household (P < 0.001), with larger households more likely to have mixed genders and 1–2 person households more likely to be adult male or adult female only. Consequently, this variable was not used in multivariable analysis and presence/absence of an adult female and presence/absence of an adult male was preferred as an indicator of gender structure of the household. When considering presence of adults in the household, presence of an adult female was significantly associated with dog ownership.

DO and DF households were compared for presence and absence of particular age categories (Table 1). Presence of the age groups 6–19 yrs, 20–29 yrs and 30–59 yrs increased the odds of owning a dog. Households where a person of 60 yrs or older was present were less likely to own a dog. Certain occupations also influenced dog ownership (Table 1). The presence of Associate Professionals, Skilled Trades and Personal Service occupations were each positively associated with dog ownership. Presence of unemployed, permanently sick/disabled persons or full-time students (including children of school age) in the household also increased the odds of dog ownership. Households with a retired person were less likely to own a dog.

Univariable analysis was also conducted on the dog owning households to compare single dog households with multiple dog households. Significant findings that increased the odds of being a multi-dog household compared to single included presence of a cat or bird (OR = 2.3, 95%CI 1.12–4.5 and OR = 4.8, 95%CI 1.7–13.5 respectively), or presence of at least one 20–29 yr old person (OR = 2.07, 95%CI 1.1–3.9).

### Hierarchical cluster analysis of age and occupation

The external validity of groups identified by cluster analysis can be assessed by comparing the results of the cluster analysis with an external criterion [28]. The age groups and occupation groups identified using hierarchical cluster analysis (described in Methods and summarised in Table 2) were both significantly associated with dog ownership in univariable analysis (P = 0.001). These household age and occupation cluster groups were used in the multivariable modelling of dog ownership instead of individual variables for each age and occupation.

### Multivariable analysis

The final model is presented in Table 3. None of the correlations between variables used in the final model were high (all <0.4). In the final model, ownership of a horse, age distribution groups, number of persons in the household, and presence of adult females were associated with the presence of one or more dogs in the household. The model appeared to fit the data well (Hosmer-Lemeshow statistic = 0.9). There were no significant two-way interactions between variables in the final model. Thirty-one (3%) households were not included in the final model due to missing data.

## Discussion

This study on dog ownership and contact with dogs focused on a small geographic area and so care is required when generalising the results to other parts of the UK or other countries. However, the percentage of the population owning a pet was almost identical to the 53% reported previously for the UK in 2004 [15], supporting the suggestion that results gained from this study may be indicative of similar populations. Dog ownership was slightly higher (24% compared to 21%), and cat ownership lower (21% compared to 25%) in the current study compared to previous estimates, which may have been due to the semi-rural location being suitable for dog ownership. In a previous American study [21], the mean number of dogs owned by dog owning households was 1.5 compared to 1.3 found here, possibly reflecting the general increased level of dog ownership in America compared to the UK (38% households versus 21–24%) and the decreasing trends for dog ownership in recent years in the UK [15]. Comparing to Australia, a study of a randomly selected group of dog owners in Perth estimated 1.2 dogs per household, similar to our findings, but again a higher percentage of all households approached were identified as owning a pet (56%) or a dog (31%) [29].

This study attempted to survey all households in a defined geographic area. In contrast, the sampling methods used in other studies, such as recruiting from veterinary, insured, internet-using, telephone-owning or dog-lover/dog-hater populations, may have introduced bias not apparent in our study method. Previous information introducing the study (leaflets), combined with the local knowledge of and community links with the local veterinary teaching hospital, may have contributed to the very good response rate for the interviews.

Univariable analysis identified a number of variables potentially associated with dog ownership including: ownership of other animals (fish, birds, horse); the presence of older children (school age); an increased number of persons in the household; Associate professional, Skilled trades and Personal service occupations, Unemployed, Permanently sick/disabled, Full-time students; and adult females. In contrast, over 60s or retired persons had lower odds of owning a dog. This may be because of reduced mobility or not replacing a deceased pet because of a new pet's perceived longevity. On further (multivariable) analysis, ownership of a horse, age distribution and number of persons in the household and presence of adult females were found to be the most important factors. Ownership of fish and birds did not remain in the final model, whereas ownership of a horse was concluded to be associated. Possibly the commitment in regards to time, care and expenses given by horse owners to their horse(s) complement the required lifestyle when owning a dog. This finding has not been reported previously but may be due to the semi-rural nature of the study area.

This study supports the suggestion that pets, in this case dogs, are more common in families who have children [16-26]. However this effect may be modified by the age of the children. In our multivariable model, families with young children (in this case five years and under) were less likely to own dogs, and similar findings have been reported by others [18,22]. In contrast, Teclaw *et al*. [20] concluded (on the basis of univariable analysis only) that there was no significant effect of young children in the household on pet ownership. Amongst young children, dog ownership is a risk factor for zoonotic disease, for example campylobacteriosis [30], however reduced dog ownership by families with young children may lessen this effect.

Several theories have been proposed to account for potential interactions between pet ownership and the presence of children in a household [5,18,31]. Our finding that dogs are often owned by households with older children could be explained if children in the older age groups had encouraged their parents to acquire a dog, and/or the parents felt that that ownership would benefit the children [18]. Alternatively, some parents may have acquired dogs as surrogate dependents [5] as their children grew up and became less receptive to physical contact and being fussed over.

In our study there was no significant effect of housing type, whereas previous work has suggested that pet owners are more likely to live in single-family dwellings and larger houses [16,17]. Such differences between studies may reflect real differences in the study population, or may be due to the fact that we were considering only dog ownership compared to general pet ownership, and/or insufficient power to detect a difference in a small and relatively homogeneous study area.

Variations in pet ownership with annual household income level [16-18], [20-23] are possibly comparable to the variations in occupations found on univariable analysis in this study. Dog ownership was associated with higher household incomes in some American studies [16,17,20,21,23]. However, the occupations indicated by our findings as being associated with dog ownership (Associate professional and technical, Skilled trade and Personal service) are not ones that would necessarily be expected to receive high incomes (for example Managers and senior officials). The role of occupation or income in dog ownership is likely to be intertwined with other factors and may be not as important as seems; this is supported by the fact that it was not significant in our final multivariable model. Similarly in another American study, stratification by household characteristics and life groups (similar to our age cluster groupings) appeared to account for the effects of education and household income on dog ownership [22]. A study in Ontario also concluded that socioeconomic status was not unconditionally associated with pet ownership after multivariable analysis [18].

The finding of presence of an adult female in the household associated with dog ownership may be due to differing attitudes to pets between the sexes. Tower and Nakota (2006) investigated the relationships between depression and pet (dogs and cats) ownership in the USA using an internet survey [26]. They found that for men: being married, living with children, being Midwestern and non-urban increased odds of living with a pet, and for women: being white, having a high income, living with children and living in a rural setting increased odds of pet ownership. They concluded that unmarried women living with a pet had the lowest depressive symptoms and unmarried men living with a pet the highest, leading them to suggest that single men may be burdened by pet ownership, whereas single women may benefit from pet companionship, but when married the pet may bring additional stress to the woman already possibly nurturing a family [26]. Our study supports the suggestion that there are underlying differences between the sexes with regard to pet ownership.

The most common reasons for dog ownership in this study (mainly "companionship") support previous research [4,9,18,31]. The elderly are a group that may be most isolated and would benefit from this companionship, as well as having something to care for and exercise [4], and yet they are less likely to own dogs compared to those people living in large families, with the most companionship already.

In our study, the reasons given for non-ownership were similar to previous findings [18] in that 'not enough time' scored highly and 'health reasons' or 'don't like dogs' scored lower, but the most common reason given in our study was 'working or being out all day' rather than 'problem when I go away' or 'housing limitations' as reported previously [18,32]. This could be due to the nature of our study area, or the use of boarding kennels possibly being a more commonplace occurrence in recent years. It must be noted that the categories given in this study were slightly different than those in previous studies. The data suggests that some of those without dogs had made a conscious decision not to own a dog (e.g. they are out all day) even though they may like to. Sixty-two percent had owned dogs in the past or lived with them at some point in their lifetime, reflecting the fact that the dog-owning population is dynamic rather than fixed. Therefore, as a person's circumstances may change, so may their risk of zoonotic disease through dog ownership.

No overall significant effect on dog ownership of cat ownership was identified. However, further analysis suggested that this relationship is more complex than first appears, as multi-dog households were significantly more likely to own a cat or bird than single dog households. It may be that some households generally have more pets, including multiple dogs, cats and birds. Interestingly multi-dog households were also more likely to contain 20–29 yr olds, possibly because young adults have the time and energy to own multiple dogs.

Clearly in this study, dog owners not only have extensive contact with their own dog, but also have increased contact with other dogs compared to those without dogs. There is a possibility that DO respondents had a greater awareness of dogs in general and this led to recall bias. The increased contact seems to be mainly through walking. It could not be determined if dog owners actually walk more or are more likely to offer walking as a reason for contacting dogs. People who own dogs may also be more likely to walk in areas frequented by other dogs, as these areas provide for socialisation of both dogs and owners, and may provide off-lead play areas which are free from hazards. Some dog owners stated employment as a reason for contacting dogs although only a small number actually worked in dog-related professions. The decreased likelihood for dog owners to report contact with a neighbour's dog may be due to recall bias. A dog owner questioned about contact may immediately identify 'walking' as a reason, whereas for non-dog owners, a neighbour's dog may be more likely to be recalled (especially if not liked).

The results of this study may be of use in behavioural research, for provision of veterinary and other services and to inform strategies for quantifying health benefits and risks associated with dog ownership. Detailed studies on the type of dog-human and dog-dog interactions that occur in the pet dog population are now needed.

## Conclusion

Some households are more likely to own a dog than others and this is associated with a number of factors, including number of people, ages of those people, an adult female in the household and ownership of a horse. Other pets, such as cats or birds, appear to be associated with multiple dog households. Dog owners also have increased contact with dogs in general (other than their own) compared with those not owning a dog, and this contact seems to be mainly through walking.

## Methods

A community of 1278 houses in Cheshire, UK, was identified as the study area. This area is on the edge of a town and was selected because it: is reasonably well defined by natural boundaries; has a mixture of medium and low-density housing; has public amenities including parks; and is near to sports fields, a wildlife reserve and agricultural land. Data were gathered using a questionnaire containing multiple choice and open-ended questions, administered during face-to-face doorstep interviews. The questionnaire had been thoroughly pre-tested, revised and piloted on approximately 100 households in a nearby area. It was designed using a high-accuracy, high-throughput automated content capture system, TELE*form *v9.1 (Verity Software, 2005), aiding design in a professional format and facilitating rapid and accurate data entry.

Each household was identified by address and visited up to five times over a five week period (July-August 2005). The time of visiting each house varied between 2 pm-8 pm weekdays and 10 am-5 pm Saturdays in an attempt to increase the possibility of interview, as identified in a pilot study. A week prior to commencement of the interviews all households were sent a leaflet to inform them of the study. Persons willing and over 16 yrs of age were interviewed on the door-step by trained interviewers following specified procedures to minimise interviewer bias. They were asked about their pets, possible reasons for not owning a dog, contact with dogs and household demographics. This included for each individual household member: gender, age category and job description or other reasons for not being in employment. Job descriptions were later categorised if possible into general types based on Standard Occupational Classification 2000 [33]. Interviewees could terminate the interview at any time or not disclose certain information if they wished and they were assured that the information would remain confidential. The interview took approximately two minutes.

Data were managed in a Microsoft Access Database and analysed using Microsoft Excel (Microsoft Corporation, 2003) Minitab (Minitab release 14.2, Minitab Inc, 2005) and SPSS (SPSS 13.0 for Windows, SPSS Inc, 2004). Dog-owning (DO) and dog-free (DF) household responses to each question were initially compared using chi-squared analysis, Fisher's exact test and univariable logistic regression. Similar methods were used to compare single with multiple dog households. When considering data collected at the level of the individual household member, the analysis was done at the household level considering presence or absence of each category.

Development of a multivariable model of dog ownership was complex due to correlation between many of the demographic variables measured at the level of the household member (age and occupation), rather than the household level (at which the outcome was measured). Because of this, hierarchical cluster analysis was used to classify households into categories by age distribution and separately into occupation categories (excluding full-time students, unemployed, looking after home/family, or permanently sick/disabled), using the Ward method for distance measurement and based on presence or absence of appropriate categories. This was an iterative process until satisfactory division of clusters was reached that approximated some real-life meaning. The age (n = 6) and occupation (n = 9) distribution cluster groupings were used to build a multivariable model of dog ownership by backward elimination. Variables and interaction terms remained in the model if they were significant in the model (P < 0.05) or if removal resulted in substantial change to the effect of other variables (10% or greater). The fit of the model was assessed using the Hosmer-Lemeshow statistic.

## Authors' contributions

CW designed the study, carried out the data collection, performed the data analysis and drafted the manuscript. RMC and GP assisted with data collection and analysis. RMC, RMG, GP, SD and JWSB conceived of the study and participated in its design and coordination and helped to draft the manuscript. All authors read and approved the final manuscript.

## Figures and Tables

**Table 1:** Univariable analysis of factors associated with dog ownership in a community in Cheshire, UK.

**Variable**	**DO**	**DF**	**OR**	**95% CI**	**P-value**
**Birds**					**0.02**
No	246	788	1		
Yes	16	24	2.1	1.1–4.1	
**Fish**					**0.02**
No	220	725	1		
Yes	42	87	1.6	1.1–2.4	
**Horse**					0.002*
No	253	807	1		
Yes	9	5	5.7	1.9–17.3	
**N° Persons**					**<0.001**
1	39	177	1		
2	85	303	1.3	0.8–1.9	
3	50	119	1.9	1.2–3.1	
4	56	160	1.6	1.0–2.5	
5+	31	41	3.4	1.9–6.1	
**Presence of 6–19 yr olds**					**0.001**
No	164	591	1		
Yes	96	209	1.7	1.2–2.2	
**Presence of 20 to 29 yr olds**					**0.03**
No	202	670	1		
Yes	58	130	1.5	1.1–2.1	
**Presence of 30 to 59 yr olds**					**<0.001**
No	61	286	1		
Yes	199	514	1.8	1.3–2.5	
**Presence of 60 yrs or above**					**0.02**
No	184	501	1		
Yes	76	299	0.7	0.5–0.9	
**Presence of Associate professional and technical**					**0.04**
No	211	691	1		
Yes	49	109	1.5	1.0–2.1	
**Presence of Skilled trades**					**0.001**
No	200	688	1		
Yes	60	112	1.8	1.3–2.6	
**Presence of Personal service**					**<0.001**
No	228	755	1		
Yes	32	45	2.4	1.5–3.8	
**Presence of Unemployed**					0.004*
No	252	795	1		
Yes	8	5	5.1	1.6–15.6	
**Presence of Retired**					**0.002**
No	193	511	1		
Yes	67	289	0.6	0.5–0.8	
**Presence of Full-time student**					**0.01**
No	163	570	1		
Yes	97	230	1.5	1.1–2.0	
**Presence of adult female**					**0.004**
No	12	84	1		
Yes	245	700	2.5	1.3–4.6	
**Adult gender household**					**0.01**
All male	12	84	1		
All female	40	144	1.9	1.00–3.9	
Mixed male/female	205	554	2.6	1.4–4.8	

* Fisher's exact test P-Value instead of Pearson Chi-Square

Significant findings on univariable analysis only reported.

**Table 2:** Description of groups from hierarchical cluster analysis of age and occupations.

**Household age categories (χ^2 ^P = 0.001)**	**Description of households**
**1 **(Over 60s)	Persons over 60 yrs present in all households, size mainly 1–2 persons.
**2 **(Families)	Very few households with under 5 yrs present, some with 6–19 yrs, many with 20–29 yrs, many with 30–59 yrs, size 1–5 persons.
**3 **(Families)	6–19 yrs present in all, 30–59 yrs in all, size 2–5 persons.
**4 **(Singles/couples adults)	30–59 yrs present in all households, size 1–4 persons (mostly 1–2).
**5 **(Young families)	Under 5 yrs present in all households, many 6–19 yrs, few 20–29 yrs, many 30–59 yrs, very few 60+yrs, size mainly 3–5 persons.
**6 **(Older families)	Very few households with 6–19 yrs present, few 20–29 yrs, many 30–59 yrs, all households 60+yrs present, size mainly 2–4 persons.

**Household occupation categories (χ^2 ^P < 0.001)**	**Description of households**

**1 **Sales	Sales occupation present in all households, other occupations mainly professionals, associate professionals, admin, process/plant, retired persons.
**2 **Skilled trade	Skilled trade occupation present in all households, other occupations mainly managers, professionals, sales persons.
**3 **Administrative and secretarial	Admin occupation present in all households, other occupations mainly managers, associate professionals, skilled, retired persons.
**4 **Retired	Retired occupation present in all households, no other occupations present.
**5 **Personal service	Personal service occupation present in all households, other occupations mainly managers, associate professionals, admin, skilled trades, sales, process/plant and retired persons.
**6 **Associate professionals	Associate professional occupation present in all households, other occupations mainly managers, professionals, skilled trade persons.
**7 **Process/plant and elementary.	Process plant and elementary occupation present, other occupations mainly associate professionals, admin, skilled trade persons.
**8 **Professional	Professional occupation present in all households, also other occupations mainly admin and retired persons.
**9 **Managers and senior officials	Manager occupation present in all households, other occupations mainly professional persons.

Key to occupations: Manager and senior officials (managers), Professional occupations (professionals), Associate professional and technical occupations (associate professionals), Administrative and secretarial occupations (Admin), Skilled trades occupations (skilled), Personal service occupations (personal service), Sales and customer service occupations (sales), Process, plant and machine operatives (process/plant), Elementary occupations (elementary).

**Table 3:** Multivariable logistic regression model of factors associated with dog ownership in a community in Cheshire (n = 1044).

**Variable**	**Coef**	**SE**	**OR**	**95% CI**	**P-value**
**Horse**					**0.005**
No	0		1		
Yes	1.6	0.6	5.1	1.7–15.5	
**Number of persons**					**0.06**
1	0		1		
2	-0.1	0.3	0.9	0.5–1.5	
3	0.3	0.4	1.3	0.6–2.8	
4	0.1	0.4	1.1	0.5–2.4	
5+	10.9	0.4	2.4	1.0–5.7	
**Age group (from cluster analysis)**					**0.04**
5 Young families	0		1		
1 Over 60's	0.4	0.4	1.5	0.7–3.5	
2 Families	0.8	0.3	2.3	1.2–4.3	
3 Families	0.8	0.3	2.2	1.2–4.0	
4 Singles/couples adults	0.8	0.4	2.3	1.0–5.2	
6 Older families	0.9	0.4	2.5	1.1–5.5	
**Presence of adult female**					**0.03**
No	0		1		
Yes	0.8	0.4	2.2	1.1–4.6	
